# Efficacy analysis of prophylactic hyperthermic intraperitoneal chemotherapy in patients with locally advanced gastric cancer: a retrospective study

**DOI:** 10.3389/fonc.2024.1503045

**Published:** 2025-01-09

**Authors:** Zhijie Gong, Liping Zhou, Yinghao He, Jun Zhou, Yanjie Deng, Zudong Huang, WeiWei Wang, Qiangbang Yang, Jian Pan, Yingze Li, Xiaolu Yuan, Minghui Ma

**Affiliations:** ^1^ Second Department of Gastrointestinal Surgery, Maoming People’s Hospital, Maoming, China; ^2^ The First School of Clinical Medicine, Southern Medical University, Guangzhou, China; ^3^ Gastrointestinal Endoscopy Center, Maoming People’s Hospital, Maoming, China; ^4^ The First Clinical College of Medicine, Guangdong Medical University, Zhanjiang, China; ^5^ Department of Pathology, Maoming People’s Hospital, Maoming, China

**Keywords:** gastric cancer, prophylactic hyperthermic intraperitoneal chemotherapy, prognosis, peritoneal metastasis, locally advanced

## Abstract

**Purpose:**

This study aims to evaluate the effectiveness and safety of prophylactic hyperthermic intraperitoneal chemotherapy (P-HIPEC) in patients with locally advanced gastric cancer (AGC) after laparoscopic radical gastrectomy. Additionally, it explores how the frequency and timing of P-HIPEC influence treatment outcomes.

**Methods:**

A retrospective analysis was conducted on 227 patients with locally AGC who underwent laparoscopic surgery at Maoming People’s Hospital from January 2016 to December 2022. Patients were stratified into the HIPEC group (n=101) and the non-HIPEC group (n=126), based on whether they received postoperative P-HIPEC. Propensity score matching (PSM) was used to adjust for baseline characteristics, facilitating a comparative analysis of survival outcomes, postoperative complications and recurrence patterns. Cox regression analysis was performed to identify prognostic factors. Furthermore, the impact of varying P-HIPEC frequencies and initiation timings was evaluated.

**Results:**

No significant differences in overall survival (OS) or postoperative complication rates were observed between the two groups in the original and PSM cohorts. But the disease-free survival (DFS) of the HIPEC group was significantly higher than that of the non-HIPEC group (HR 0.569; 95% CI 0.362–0.894; *p =* 0.013) in the PSM cohort, with 1-year, 3-year, and 5-year DFS rates showing notable improvement (77.9% vs. 69.7%, 60.1% vs. 43.0%, and 46.2% vs. 25.5%). The incidence of isolated peritoneal metastasis (PM) was significantly lower in the HIPEC group (5.3% vs. 17.3%, *p =* 0.039). Multivariate Cox regression analysis identified P-HIPEC as an independent protective factor for DFS. Further analysis indicated that neither the number of P-HIPEC sessions had a significant impact on OS (*p =* 0.388) or DFS (*p =* 0.735), nor did the timing of P-HIPEC initiation affect OS (*p =* 0.620) or DFS (*p =* 0.488). Likewise, different P-HIPEC frequencies or initiation timings had no significant impact on postoperative complication rates or recurrence patterns.

**Conclusion:**

P-HIPEC effectively reduces the risk of postoperative PM and improves DFS in patients with locally AGC without increasing postoperative complications. However, it does not significantly impact OS. Additionally, variations in the frequency and timing of P-HIPEC initiation do not significantly affect survival outcomes, postoperative complications, or recurrence patterns.

## 1 Introduction

Gastric cancer ranks as the fifth most prevalent malignancy globally and is the third primary cause of cancer-related fatalities (1). Early-stage gastric cancer often presents without symptoms, resulting in many patients being diagnosed at an advanced stage (2). For locally AGC, radical resection with D2 lymphadenectomy combined with systemic chemotherapy constitutes the standard treatment approach. However, therapeutic outcomes remain unsatisfactory, with nearly 50% of patients developing peritoneal metastasis (PM) despite receiving standard treatment (3). Once PM occurs, the median survival drops to just 4.6 months, and the 5-year survival rate is nearly zero (4). Thus, reducing postoperative PM and improving prognosis in patients with locally AGC remains a critical focus for future research.

Hyperthermic intraperitoneal chemotherapy (HIPEC) involves the perfusion of a heated chemotherapy solution into the abdominal cavity under controlled pressure. By combining the synergistic effects of hyperthermia and chemotherapy with mechanical lavage, HIPEC aims to eradicate or suppress cancer cells and micrometastases within the peritoneal cavity (5, 6). For various peritoneal malignancies, such as malignant peritoneal mesothelioma and pseudomyxoma peritonei, cytoreductive surgery (CRS) combined with HIPEC has become the cornerstone treatment for extending patient survival (7, 8). In gastric cancer, CRS + HIPEC has demonstrated an enhancement in the prognosis of patients with AGC and concurrent PM (9–11). However, for locally AGC, the postoperative application of prophylactic HIPEC (P-HIPEC) remains a subject of debate. Some studies have reported P-HIPEC has no impact on patients’ Disease-Free Survival (DFS) or Overall Survival (OS) (12, 13). Conversely, some other research indicate that P-HIPEC may significantly improve DFS (14, 15). In addition, there is no clear consensus on the ideal frequency and initiation timing of P-HIPEC, and the available data are limited.

This retrospective study assesses the efficacy and safety of P-HIPEC in patients with locally AGC after laparoscopic radical gastrectomy. Additionally, it explores the effects of different P-HIPEC frequencies and initiation timings on patient prognosis and postoperative complications.

## 2 Materials and methods

### 2.1 Patients and design

This study is a retrospective cohort analysis of patients diagnosed with locally AGC who underwent laparoscopic surgery at Maoming People’s Hospital between January 2016 and December 2022. The inclusion criteria were as follows: (1) age between 18 and 75 years; (2) ECOG performance status of 0-1; (3) postoperative pathological confirmation of T3-T4 primary gastric cancer, with or without lymph node metastasis(N0-N3); (4) preoperative imaging evaluation reveals no distant metastasis(M0) and no visible peritoneal metastasis (PM) during surgery; and (5) no history of prior neoadjuvant radiotherapy or chemotherapy. The exclusion criteria were as follows: (1) conversion from laparoscopic to open surgery (n=11); (2) positive tumor margins (n=9); (3) emergency surgery (n=5); (4) remnant gastric cancer (n=13); and (5) loss to follow-up after surgery (n=17). Ultimately, 227 patients met the inclusion criteria and were included in the analysis. The consultation times of the patients included in the study were evenly distributed between 2016 and 2022, with no differences in the time dimension between the two groups.

**Figure 1:**
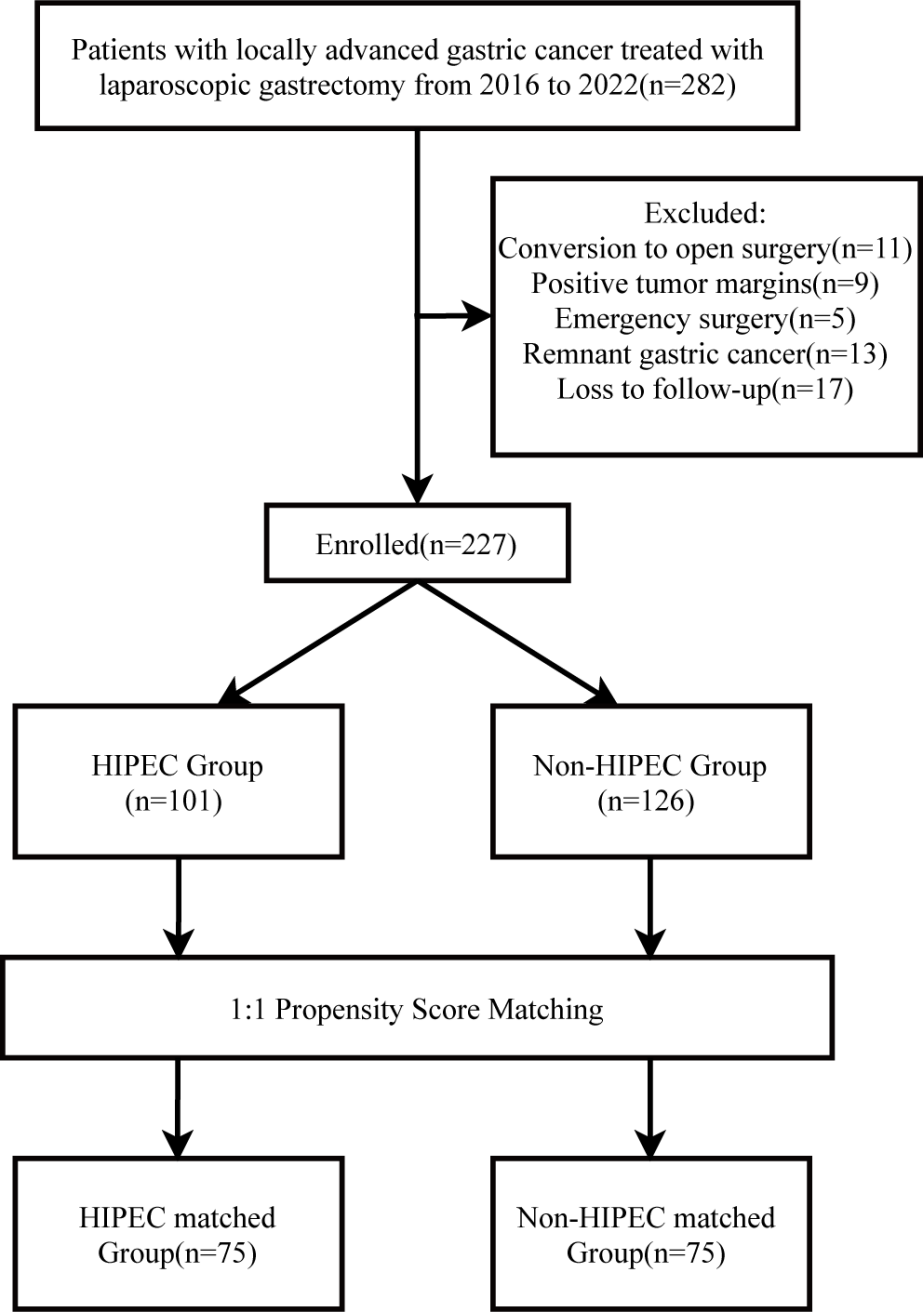
The flowchart of this study.

Patients were divided into two groups based on whether they received postoperative P-HIPEC: the HIPEC group (n=101) and the non-HIPEC group (n=126). To reduce selection bias and balance baseline clinical characteristics between the two groups, propensity score matching (PSM) was performed. Matching was conducted at a 1:1 ratio using a caliper width of 0.2, resulting in 75 patients in each group (HIPEC-matched group, n=75; non-HIPEC-matched group, n=75). The study flowchart is shown in **Figure 1**. This study was approved by the Ethics Committee of Maoming People’s Hospital (approval number: PJ2020MI-K183-01). The requirement for individual informed consent for the study was waived because the research involved only anonymized retrospective data.

### 2.2 Treatment

All 227 patients received laparoscopic surgery conducted by the same team of proficient gastrointestinal surgeons. Laparoscopic exploration was first conducted during surgery to ensure no visible peritoneal metastasis (PM) nodules. Preoperative peritoneal lavage cytology was performed for some patients, and those with positive results were excluded from this study. The majority of patients underwent distal or total gastrectomy based on the location of tumor. For a small number of patients with tumors in the upper stomach who requested stomach preservation and refused total gastrectomy, proximal gastrectomy was performed following preoperative assessment and multidisciplinary team discussion. All patients underwent standard D2 lymphadenectomy. The reconstruction procedure differed according to the extent of gastrectomy and comprised Billroth I gastroduodenostomy, Billroth II gastrojejunostomy, Roux-en-Y esophagojejunostomy, and esophagogastrostomy.

The decision to proceed with P-HIPEC is made collaboratively by the patient and their family, after they have been fully informed of the potential risks and benefits, with the understanding that they can choose to discontinue treatment at any point during the cycle. Based on the ‘Expert Consensus on Clinical Application of Intraperitoneal Hyperthermic Perfusion Chemotherapy Technology’ (2016 edition, in Chinese) and our institutional experience, we typically recommend initiating HIPEC within one to two days postoperatively and performing up to 3–5 HIPEC treatments, with intervals between sessions usually of 24 hours but extendable to 48 hours if necessary. In actual practice, the initiation timing and frequency of HIPEC were primarily determined by the patients’ postoperative recovery and tolerance levels.

The chemotherapy agents primarily used were oxaliplatin (80–160 mg) and fluorouracil (0.5–0.75 g). For patients undergoing multiple HIPEC sessions, oxaliplatin was administered only during the first session to reduce the risk of drug toxicity, with subsequent sessions using fluorouracil alone. Chemotherapy medications were dissolved in 500 mL of saline and combined with 3000 mL of regular saline to create the perfusion solution. Using the BR-TRG-II Intraperitoneal Hyperthermic Perfusion System (manufactured by Guangzhou Baorui Medical Equipment Co., Ltd., Guangzhou, China), the chemotherapy solution was heated to 42–43°C and infused into the patient’s abdominal cavity. The flow rate was sustained at 500-600 mL/min via a perfusion pump, and the solution circulated within the abdominal cavity for 60 minutes to ensure high local drug concentration exposure to the peritoneum. Parecoxib sodium or flurbiprofen axetil was administered intravenously to alleviate pain. During the entire perfusion process, core body temperature, abdominal temperature, and hemodynamic status were continuously monitored to ensure precise control. After HIPEC, the perfusion fluid was drained.

In this study, patients who received 1 or 2 HIPEC sessions were classified as the low-frequency HIPEC group, while those who received 3 to 5 sessions were classified as the high-frequency HIPEC group. HIPEC initiated on the day of surgery or within 1 to 2 days postoperatively was defined as early HIPEC, whereas HIPEC starting on postoperative day three or later was defined as late HIPEC.

All patients with locally AGC were advised to undergo at least six cycles of systemic chemotherapy, using either the SOX or XELOX regimens, starting 4–6 weeks postoperatively. To account for the impact of inadequate chemotherapy on patient prognosis (16, 17), patients who received no more than two cycles of systemic chemotherapy were categorized into the non-chemotherapy group during the baseline characteristics analysis.

### 2.3 Follow-up

Patients were followed up every three months for the initial two years post-surgery, every six months from years three to five, and annually thereafter. Follow-up was conducted through outpatient visits and telephone calls. Assessments such as contrast enhanced chest and abdominal CT scans, tumor marker tests, and gastroscopy were performed during outpatient visits. Telephone follow-ups were performed for patients who could not attend outpatient appointments. The follow-up period ended in May 2024. The principal outcomes were OS, defined as the duration from surgery to death, and DFS, defined as the interval from surgery to tumor recurrence or death.

### 2.4 Statistical analysis

All variables were presented as frequencies and percentages, and group differences were evaluated using the Chi-square test or Fisher’s exact test. Kaplan-Meier survival curves were constructed to visualize survival distributions, and the Log-rank test was employed to evaluate differences in survival rates. To further analyze factors affecting OS and DFS, a univariate Cox regression analysis was conducted for each variable. Variables exhibiting a *p-*value < 0.1 in the univariate analysis were subsequently incorporated into a multivariate Cox proportional hazards model to ascertain independent prognostic factors. All analyses were conducted using R version 4.3.3 (https://www.r-project.org/), with *p*-values < 0.05 considered statistically significant.

## 3 Results

### 3.1 Patient characteristics

In the original cohort, 227 patients were included, with 126 in the non-HIPEC group and 101 in the HIPEC group. **Table 1** presents the baseline characteristics of the two groups. The clinical characteristics, including gender, age, nutrition score (NRS2002), ECOG score, tumor markers (CEA and CA199), tumor invasion depth, pathological type, degree of differentiation, vascular and perineural invasion, along with tumor location and size, were generally comparable (*P* > 0.05). However, notable disparities were identified in some variables. The distribution of N stage differed significantly (*P =* 0.009), with the non-HIPEC group having a higher proportion of patients in N1 (29.4% vs. 17.9%) and N3 stages (25.4% vs. 17.8%). Additionally, the HIPEC group had a higher proportion of patients with larger tumors (>5 cm) compared to the non-HIPEC group (46.5% vs. 32.5%, *P =* 0.044), while the non-HIPEC group had a higher incidence of hypertension (22.2% vs. 10.9%, *P =* 0.038). After PSM, 75 patients were included in each group, with all covariates balanced (*P* > 0.05) (**Table 1**).

**Table 1:** The characteristics of patients before and after PSM.

Variables	Before PSM			After PSM		
	Non-HIPEC	HIPEC	*P*	Non-HIPEC	HIPEC	*P*
	(n=126)	(n=101)		(n=75)	(n=75)	
Gender			*0.360*			*0.867*
Female	51 (40.5%)	34 (33.7%)		30 (40%)	28 (37.3%)	
Male	75 (59.5%)	67 (66.3%)		45 (60%)	47 (62.7%)	
Age(years)			*0.443*			*1*
<60	45 (35.7%)	42 (41.6%)		29 (38.7%)	30 (40%)	
≥60	81 (64.3%)	59 (58.4%)		46 (61.3%)	45 (60%)	
Nutrition score			*0.909*			*0.824*
≤2	108 (85.7%)	88 (87.1%)		62 (82.7%)	64 (85.3%)	
≥3	18 (14.3%)	13 (12.9%)		13 (17.3%)	11 (14.7%)	
ECOG score			*0.455*			*0.440*
0	96 (76.2%)	82 (81.2%)		55 (73.3%)	60 (80%)	
1	30 (23.8%)	19 (18.8%)		20 (26.7%)	15 (20%)	
Comorbidity						
Diabetes	14 (11.1%)	10 (9.9%)	*0.938*	8 (10.7%)	6 (8%)	*0.779*
Hypertension	28 (22.2%)	11 (10.9%)	*0.038*	11 (14.7%)	9 (12%)	*0.810*
Hemoglobin			*0.372*			*0.620*
Anemia	61 (48.4%)	42 (41.6%)		30 (40%)	34 (45.3%)	
Normal	65 (51.6%)	59 (58.4%)		45 (60%)	41 (54.7%)	
CEA			*0.624*			*1*
Negative	97 (77%)	74 (73.3%)		57 (76%)	57 (76%)	
Positive	29 (23%)	27 (26.7%)		18 (24%)	18 (24%)	
CA199			*0.621*			*0.481*
Negative	91 (72.2%)	69 (68.3%)		49 (65.3%)	54 (72%)	
Positive	35 (27.8%)	32 (31.7%)		26 (34.7%)	21 (28%)	
pT stage			*0.152*			*0.714*
T3	42 (33.3%)	24 (23.8%)		22 (29.3%)	19 (25.3%)	
T4	84 (66.7%)	77 (76.2%)		53 (70.7%)	56 (74.7%)	
pN stage			*0.009*			*0.871*
N0	37 (29.4%)	15 (14.9%)		17 (22.7%)	14 (18.7%)	
N1	26 (20.6%)	30 (29.7%)		21 (28%)	24 (32%)	
N2	31 (24.6%)	38 (37.6%)		20 (26.7%)	22 (29.3%)	
N3	32 (25.4%)	18 (17.8%)		17 (22.7%)	15 (20%)	
Histology			*0.502*			*0.484*
Adenocarcinoma	106 (84.1%)	90 (89.1%)		65 (86.7%)	69 (92%)	
Signet	16 (12.7%)	8 (7.9%)		7 (9.3%)	5 (6.7%)	
Mucinous	4 (3.2%)	3 (3%)		3 (4%)	1 (1.3%)	
Differentiation			*0.249*			*0.699*
Poorly	97 (77%)	70 (69.3%)		59 (78.7%)	56 (74.7%)	
Well/Moderately	29 (23%)	31 (30.7%)		16 (21.3%)	19 (25.3%)	
Vascular invasion			*0.767*			*0.510*
No	32 (25.4%)	25 (24.8%)		21 (28%)	15 (20%)	
Yes	86 (68.3%)	67 (66.3%)		49 (65.3%)	55 (73.3%)	
Unknown	8 (6.3%)	9 (8.9%)		5 (6.7%)	5 (6.7%)	
Perineural invasion			*0.397*			*0.759*
No	34 (27%)	20 (19.8%)		19 (25.3%)	16 (21.3%)	
Yes	77 (61.1%)	70 (69.3%)		50 (66.7%)	51 (68%)	
Unknown	15 (11.9%)	11 (10.9%)		6 (8%)	8 (10.7%)	
Tumor location			*0.384*			*0.944*
Antrum	70 (55.6%)	56 (55.4%)		44 (58.7%)	42 (56%)	
Body	42 (33.3%)	28 (27.7%)		20 (26.7%)	21 (28%)	
Cardia	14 (11.1%)	17 (16.8%)		11 (14.7%)	12 (16%)	
Tumor size(cm)			*0.044*			*0.741*
<5	85 (67.5%)	54 (53.5%)		42 (56%)	45 (60%)	
≥5	41 (32.5%)	47 (46.5%)		33 (44%)	30 (40%)	
Gastrectomy			*0.308*			*1*
Distal	82 (65.1%)	74 (73.3%)		54 (72%)	54 (72%)	
Total	36 (28.6%)	24 (23.8%)		18 (24%)	18 (24%)	
Proximal	8 (6.3%)	3 (3%)		3 (4%)	3 (4%)	
Chemotherapy			*0.421*			*1*
No	59 (46.8%)	41 (40.6%)		31 (41.3%)	30 (40%)	
Yes	67 (53.2%)	60 (59.4%)		44 (58.7%)	45 (60%)	

HIPEC, hyperthermic intraperitoneal chemotherapy; PSM, propensity score matching; CEA, carcinoembryonic antigen; CA-199, carbohydrate antigen 199.

Baseline clinical characteristics were also compared based on HIPEC frequency and initiation timing (**Supplementary Table 1**). The low-frequency HIPEC group had a significantly higher proportion of T3 stage patients compared to the high-frequency group (32.8% vs. 11.6%, *P =* 0.026). Additionally, the late HIPEC group had a lower proportion of hypertensive patients than the early HIPEC group (0% vs. 15.9%, *P =* 0.040). Aside from these differences, no other covariates were significantly different, and the groups were generally well-balanced for further comparisons.

### 3.2 Treatment results

All enrolled patients successfully underwent laparoscopic radical gastrectomy, with R0 resection achieved in all cases. Seven patients required multi-visceral resection due to tumor invasion. In the HIPEC group, two patients underwent combined transverse colon resection. In the non-HIPEC group, two patients underwent splenectomy, and three underwent combined transverse colon resection. No surgery-related deaths or intraoperative complications occurred in either group.

**Figure 2:**
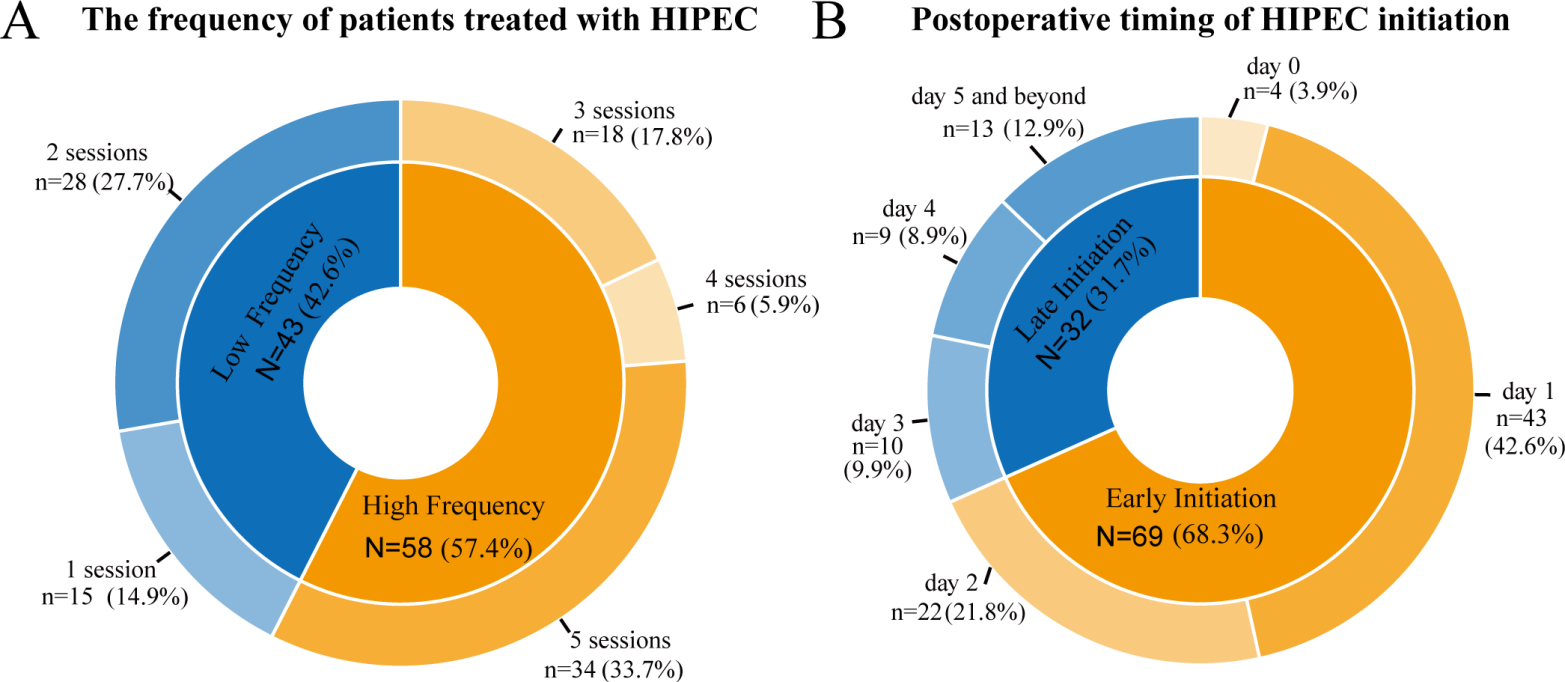
Distribution of patients based on the number of HIPEC sessions **(A)** and the timing of HIPEC initiation postoperatively **(B)**.

Regarding the chemotherapy agents used for HIPEC, 95 patients received a combination of oxaliplatin and fluorouracil, 4 were treated with fluorouracil alone, and 2 received cisplatin combined with epirubicin. The differences in the number of HIPEC sessions and the timing of HIPEC initiation are shown in **Figure 2**. More than half of patients (57.4%) underwent multiple treatment sessions, with five sessions being the most common (33.7%). As for the timing of HIPEC initiation, 68.3% of patients began treatment within the first two postoperative days or on the day of surgery, with 42.6% starting on the first postoperative day. Overall, the data indicates that most patients initiated HIPEC early and received multiple treatment sessions.

### 3.3 Survival analysis

**Figure 3:**
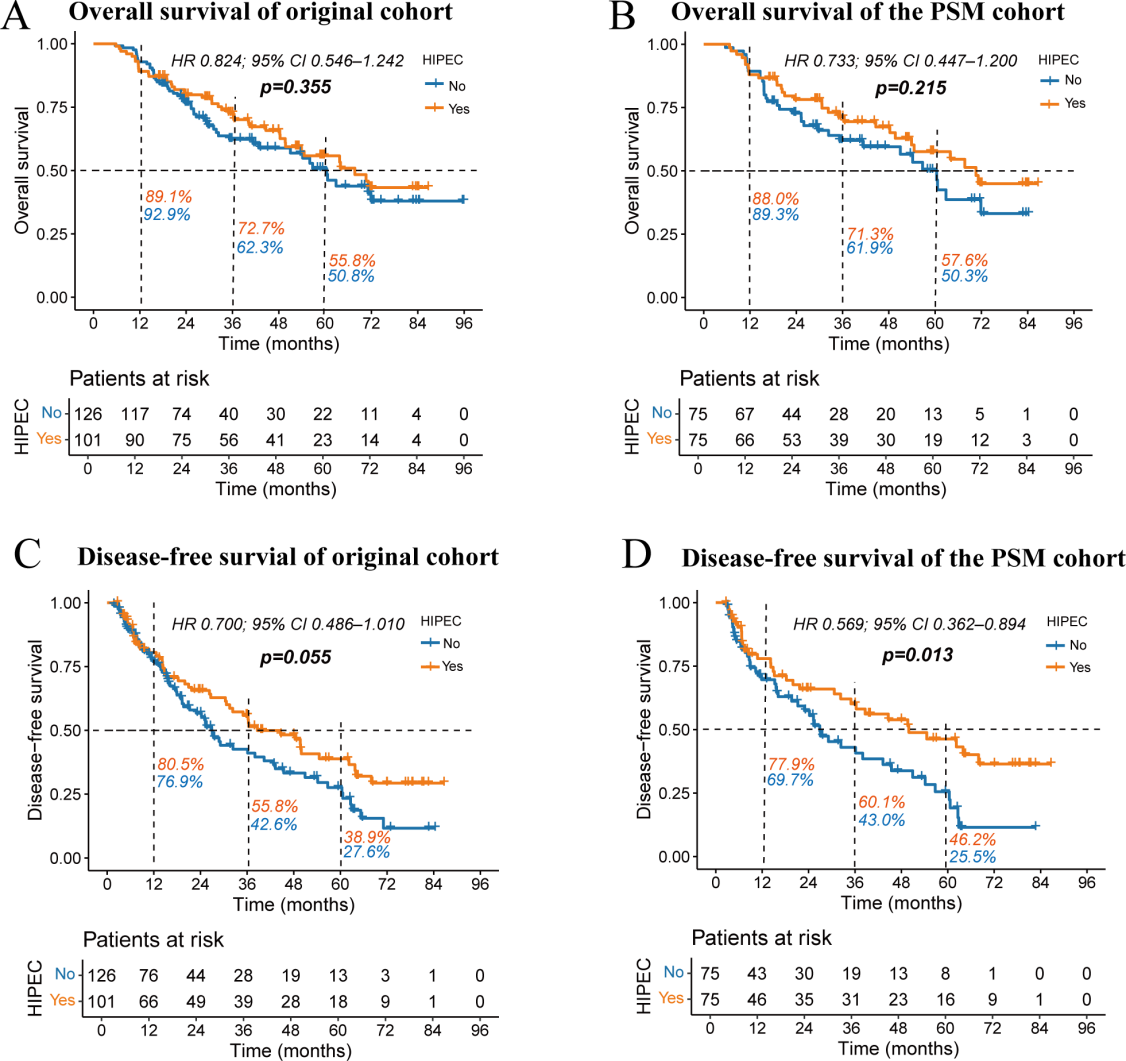
Kaplan-Meier survival curves comparing overall survival **(A, B)** and disease-free survival **(C, D)** between the HIPEC and non-HIPEC groups. The percentages next to the dashed lines represent the 1-year, 3-year, and 5-year OS or DFS rates for the two groups of patients.

The median follow-up duration was 31 months (ranging from 6 to 96 months). In the follow-up period, 51 patients (40.4%) from the non-HIPEC group and 42 patients (41.5%) from the HIPEC group died. As shown in **Figures 3A, B**, the Kaplan-Meier curves indicated no statistically significant difference in OS between the HIPEC and non-HIPEC groups in the original cohort (HR 0.824; 95% CI 0.546–1.242; *p =* 0.355). Similarly, no significant difference in OS was observed in the PSM cohort (HR 0.733; 95% CI 0.447–1.200; *p =* 0.215).

However, in the analysis of DFS (**Figures 3C, D**), the original cohort showed a trend toward improved DFS in the HIPEC group, approaching statistical significance (HR 0.700; 95% CI 0.486–1.010; *p =* 0.055). In contrast, the PSM cohort demonstrated a significantly better DFS in the HIPEC group (HR 0.569; 95% CI 0.362–0.894; *p =* 0.013). In the PSM cohort, the median DFS time was 49.9 months for the HIPEC group, compared to 26.9 months for the non-HIPEC group. The 1-year, 3-year, and 5-year DFS rates were consistently higher in the HIPEC group (77.9% vs. 69.7%; 60.1% vs. 43.0%; and 46.2% vs. 25.5%).

**Figure 4:**
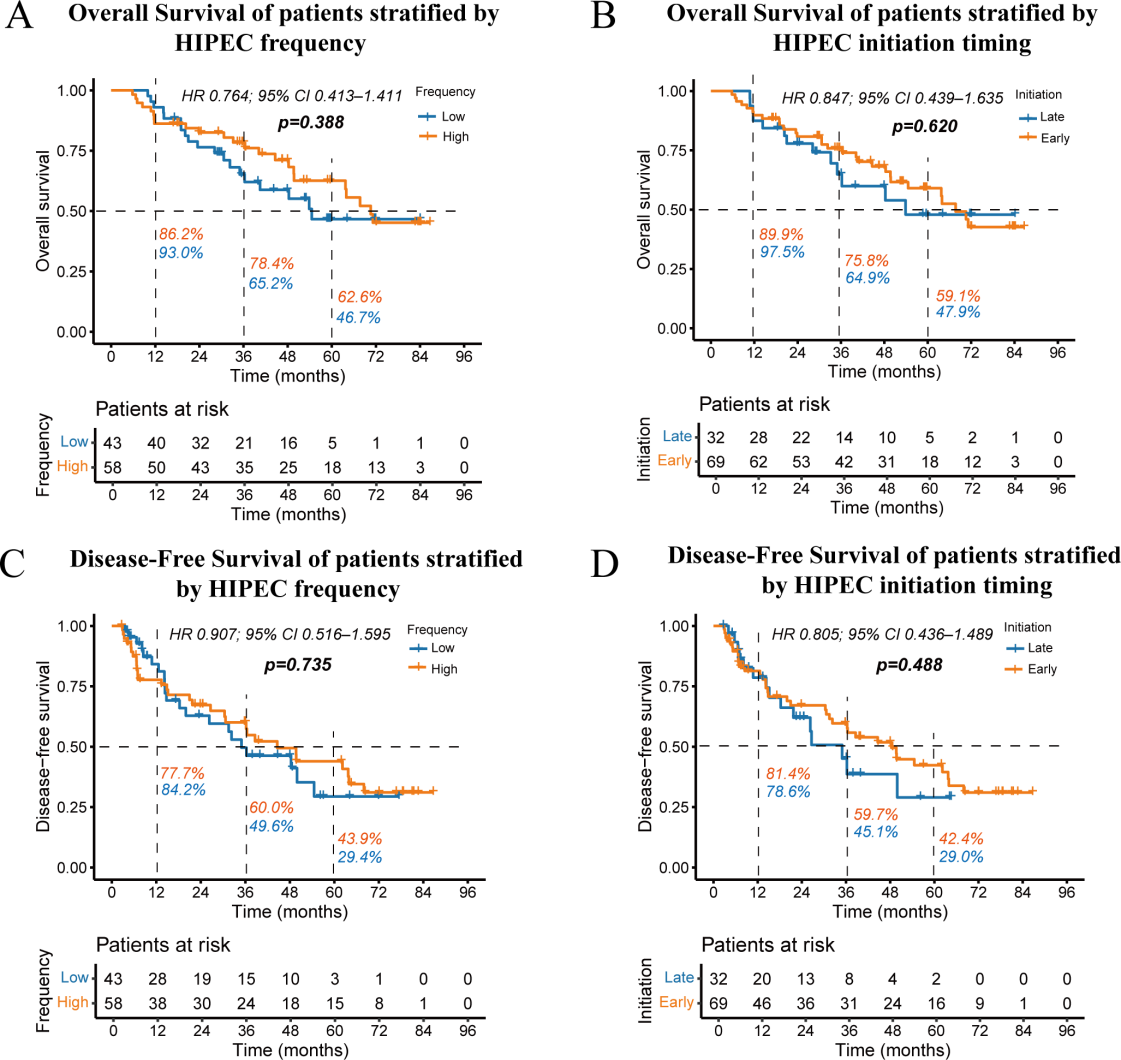
Kaplan-Meier curves showing overall survival stratified by HIPEC frequency **(A)** and initiation timing **(B)**, as well as disease-free survival stratified by HIPEC frequency **(C)** and initiation timing **(D)**. The percentages next to the dashed lines represent the 1-year, 3-year, and 5-year OS or DFS rates for the two groups of patients.

In addition, we explored the impact of HIPEC frequency and initiation timing on patient prognosis. The frequency of HIPEC treatment (low vs. high) had no significant effect on OS (*p =* 0.388) or DFS (*p =* 0.735) (**Figures 4A, C**). Similarly, the timing of HIPEC initiation (early vs. late) demonstrated no statistically significant changes in OS (*p =* 0.620) or DFS (*p =* 0.488) (**Figures 4B, D**). Overall, neither the frequency nor the initiation timing of HIPEC had a notable impact on survival outcomes in this study population.

### 3.4 Postoperative outcomes

The impact of HIPEC on postoperative complications and recurrence patterns is summarized in **Table 2**. We evaluated common postoperative complications, including anastomotic leakage, bowel obstruction, postoperative gastric paresis, wound infection, pulmonary infection, urinary tract infection, and pleural effusion. In both the original and PSM cohorts, no substantial variations were observed in the incidence of these complications between the two groups (*p* > 0.05). Regarding recurrence patterns, the original cohort showed that the non-HIPEC group (15.1%) had a greater risk of isolated PM than the HIPEC group (6.9%), but the difference did not reach statistical significance (*p =* 0.088). In the PSM cohort, the non-HIPEC group had a markedly greater incidence of isolated PM (17.3%) compared to the HIPEC group (5.3%) (*p =* 0.039). Other recurrence patterns, including isolated extraperitoneal and peritoneal and extraperitoneal recurrence, revealed no appreciable variations between the two groups.

**Table 2:** Comparison of postoperative complications and recurrence in non-HIPEC and HIPEC groups.

Variables	Original cohort			The PSM cohort		
	Non-HIPEC	HIPEC	*P*	Non-HIPEC	HIPEC	*P*
	(n=126)	(n=101)		(n=75)	(n=75)	
Complications						
Anastomotic leaks	5(4.0%)	2(2.0%)	*0.466*	3(4.0%)	1(1.35%)	*0.620*
Bowel obstruction	9(7.1%)	10(10.0%)	*0.614*	7(9.3%)	6(8.0%)	*1*
Gastroparesis	4(3.2%)	2(2.0%)	*0.695*	3(4.0%)	1(1.35%)	*0.620*
Wound infection	3(2.4%)	2(2.0%)	*1*	2(2.7%)	1(2.7%)	*1*
Lung infection	7(5.5%)	8(8.0%)	*0.657*	6(8.0%)	6(9.3%)	*1*
Urinary tract infection	2(1.6%)	2(2.0%)	*1*	1(2.7%)	2(2.7%)	*1*
Pleural effusion	3(2.4%)	1(1.0%)	*0.631*	3(4.0%)	1(2.7%)	*0.620*
Recurrence						
Isolated peritoneal	19(15.1%)	7(6.9%)	*0.088*	13(17.3%)	4(5.3%)	*0.039*
Isolated extraperitoneal	15(11.9%)	20(19.8%)	*0.146*	11(14.7%)	17(22.7%)	*0.295*
Peritoneal and extraperitoneal	15(11.9%)	9(8.9%)	*0.608*	8(10.7%)	3(4.00%)	*0.210*

HIPEC, hyperthermic intraperitoneal chemotherapy; PSM, propensity score matching.

Additionally, **Table 3** shows the impact of HIPEC frequency and initiation timing on postoperative complications and recurrence. Neither HIPEC frequency (low vs. high) nor initiation timing (early vs. late) had a statistically significant effect on these outcomes (*p* > 0.05).

**Table 3:** Impact of HIPEC frequency and timing on postoperative complications and recurrence.

Variables	HIPEC Treatment Frequency			HIPEC Initiation Timing		
	Low	High	*P*	Early	Late	*P*
	(n=43)	(n=58)		(n=69)	(n=32)	
Complications						
Anastomotic leaks	2(4.7%)	0	*0.179*	2(2.9%)	0	*1*
Bowel obstruction	6(14.0%)	4(6.9%)	*0.317*	7(10.1%)	3(9.4%)	*1*
Gastroparesis	1(2.3%)	1(1.7%)	*1*	2(2.9%)	0	*1*
Wound infection	0	2(3.4%)	*0.506*	0	2(6.3%)	*0.098*
Lung infection	4(9.3%)	4(6.9%)	*0.720*	5(7.2%)	3(9.4%)	*0.705*
Urinary tract infection	1(2.3%)	1(1.7%)	*1*	1(1.4%)	1(3.1%)	*0.535*
Pleural effusion	0	1(1.7%)	*1*	1(1.4%)	0	*1*
Recurrence						
Isolated peritoneal	2(4.7%)	5(8.6%)	*0.696*	4(5.8%)	3(9.4%)	*0.676*
Isolated extraperitoneal	6(14.0%)	14(24.1%)	*0.309*	16(23.2%)	4(12.5%)	*0.324*
Peritoneal and extraperitoneal	5(11.6%)	4(6.9%)	*0.490*	5(7.3%)	4(12.5%)	*0.459*

HIPEC, hyperthermic intraperitoneal chemotherapy; PSM, propensity score matching.

### 3.5 Univariate and multivariate analyses

**Table 4** displays the results of the univariate and multivariate Cox regression analysis for OS. In the univariate analysis, factors such as CEA, CA199, pT stage, pN stage, vascular invasion, perineural invasion, tumor size, extent of gastrectomy, and systemic chemotherapy were markedly correlated with OS. The multivariate analysis identified CA199 positivity(HR = 1.672, 95% CI: 1.053–2.656, *p =* 0.029), vascular invasion (HR = 2.181, 95% CI: 1.063–4.477, *p =* 0.034), perineural invasion (HR = 3.698, 95% CI: 1.511–9.048, *p =* 0.004), and total gastrectomy (HR = 2.037, 95% CI: 1.278–3.248, *p =* 0.004) as independent risk factors for OS, whereas systemic chemotherapy (HR = 0.626, 95% CI: 0.400–0.980, *p =* 0.041) was recognized as an independent protective factor. The administration of HIPEC, as well as the frequency and initiation timing of HIPEC, showed no significant association with OS.

**Table 4:** Univariate analysis and multivariate analysis of overall survival.

	Univariate analysis		Multivariate analysis	
Variable	HR (95% CI)	*P*	HR (95% CI)	*P*
Gender (Male/Female)	0.912(0.598-1.392)	*0.669*	−	*−*
Age(<60/≥60,years)	0.966(0.631-1.480)	*0.875*	−	*−*
Nutrition score(≤2/≥3)	0.623(0.322-1.204)	*0.159*	−	*−*
ECOG score (0/1)	0.831(0.507-1.364)	*0.464*	−	*−*
Comorbidity (Yes/No)	1.073(0.659-1.747)	*0.777*	−	*−*
Hemoglobin (Normal/Anemia)	0.841(0.560-1.265)	*0.406*	−	*−*
CEA(Positive/Negative)	1.568(1.015-2.423)	*0.043*	1.348(0.848-2.145)	*0.207*
CA199(Positive/Negative)	1.552(1.022-2.356)	*0.039*	1.672(1.053-2.656)	*0.029*
pT stage(T4/T3)	1.829(1.081-3.096)	*0.024*	1.414(0.824-2.424)	*0.209*
pN stage(N1-N3/N0)	1.828(1.051-3.177)	*0.033*	1.050(0.593-1.861)	*0.866*
Histology (Signet or Mucinous/Adenocarcinoma)	1.346(0.761-2.378)	*0.307*	−	*−*
Differentiation (Poorly/Well or moderately)	1.386(0.864-2.223)	*0.176*	−	*−*
Vascular invasion (Yes/No)	4.070(2.098-7.893)	*<0.001*	2.181(1.063-4.477)	*0.034*
Perineural invasion (Yes/No)	6.414(2.791-14.74)	*<0.001*	3.698(1.511-9.048)	*0.004*
Tumor location (Body/Antrum)	1.165(0.735-1.848)	*0.516*	−	*−*
Tumor location (Cardia//Antrum)	1.367(0.776-2.409)	*0.279*	−	*−*
Tumor size(<5/≥5,cm)	0.431(0.286-0.651)	*<0.001*	0.663(0.429-1.023)	*0.063*
Gastrectomy (Total/Distal or Proximal)	1.996(1.304-3.055)	*0.002*	2.037(1.278-3.248)	*0.003*
Chemotherapy (Yes/No)	0.498(0.327-0.758)	*0.001*	0.626(0.400-0.980)	*0.041*
HIPEC(Yes/No)	0.824(0.546-1.242)	*0.355*	−	*−*
HIPEC Frequency (Low/High)	0.665(0.333-1.326)	*0.247*	−	*−*
HIPEC Initiation (Late/Early)	1.203(0.574-2.524)	*0.624*	−	*−*

*HIPEC* hyperthermic intraperitoneal chemotherapy; *PSM* propensity score matching; *CEA* carcinoembryonic antigen; *CA-199* carbohydrate antigen 199; *HR* hazard ratio.

**Table 5** presents the results of univariate and multivariate analyses for DFS. In the univariate analysis, factors such as CEA, CA199, pN stage, degree of differentiation, vascular invasion, perineural invasion, tumor size, and extent of gastrectomy were significantly associated with DFS. In the multivariate analysis, CEA positivity (HR = 1.602, 95% CI: 1.068–2.404, *p =* 0.023), vascular invasion (HR = 1.909, 95% CI: 1.080–3.375, *p =* 0.026), perineural invasion (HR = 2.362, 95% CI: 1.163–4.797, *p =* 0.017), and total gastrectomy (HR = 1.638, 95% CI: 1.069–2.512, *p =* 0.024) were identified as independent risk factors for DFS. In contrast, undergoing HIPEC (HR = 0.660, 95% CI: 0.437–0.995, *p =* 0.047) was found to be an independent protective factor. The frequency and initiation timing of HIPEC were not associated with DFS.

**Table 5:** Univariate analysis and multivariate analysis of Disease-Free survival.

	Univariate analysis		Multivariate analysis	
Variable	HR (95% CI)	*P*	HR (95% CI)	*P*
Gender (Male/Female)	0.808(0.559-1.170)	*0.259*	−	*−*
Age(<60/≥60,years)	0.990(0.681-1.440)	*0.960*	−	*−*
Nutrition score(≤2/≥3)	0.679(0.392-1.169)	*0.162*	−	*−*
ECOG score (0/1)	0.862(0.561-1.324)	*0.498*	−	*−*
Comorbidity (Yes/No)	1.240(0.811-1.895)	*0.321*	−	*−*
Hemoglobin (Normal/Anemia)	0.951(0.664-1.363)	*0.785*	−	*−*
CEA(Positive/Negative)	1.604(1.094-2.351)	*0.016*	1.602(1.068-2.404)	*0.023*
CA199(Positive/Negative)	1.469(1.011-2.135)	*0.044*	1.306(0.861-1.982)	*0.209*
pT stage(T4/T3)	1.535(0.980-2.403)	*0.061*	1.076(0.675-1.716)	*0.757*
pN stage(N1-N3/N0)	1.852(1.121-3.059)	*0.016*	1.398(0.819-2.386)	*0.220*
Histology (Signet or Mucinous/Adenocarcinoma)	1.458(0.869-2.447)	*0.154*	−	*−*
Differentiation (Poorly/Well or moderately)	1.529(1.005-2.326)	*0.047*	1.235(0.796-1.915)	*0.346*
Vascular invasion (Yes/No)	2.900(1.726-4.873)	*<0.001*	1.909(1.080-3.375)	*0.026*
Perineural invasion (Yes/No)	3.694(1.926-7.083)	*<0.001*	2.362(1.163-4.797)	*0.017*
Tumor location (Body/Antrum)	1.166(0.775-1.754)	*0.462*	−	*−*
Tumor location (Cardia//Antrum)	1.357(0.833-2.211)	*0.220*	−	*−*
Tumor size(<5/≥5,cm)	0.656(0.458-0.940)	*0.022*	0.810(0.556-1.178)	*0.270*
Gastrectomy (Total/Distal or Proximal)	1.706(1.166-2.496)	*0.006*	1.638(1.069-2.512)	*0.024*
Chemotherapy (Yes/No)	0.772(0.538-1.108)	*0.161*	−	*−*
HIPEC(Yes/No)	0.701(0.486-1.010)	*0.057*	0.660(0.437-0.995)	*0.047*
HIPEC Frequency (Low/High)	0.655(0.348-1.233)	*0.189*	−	*−*
HIPEC Initiation Timing (Late/Early)	1.269(0.646-2.493)	*0.490*	−	*−*

HIPEC, hyperthermic intraperitoneal chemotherapy; PSM, propensity score matching; CEA, carcinoembryonic antigen; CA-199, carbohydrate antigen 199; HR, hazard ratio.

## 4 Discussion

In recent years, laparoscopic surgery has achieved broad acceptability for the treatment of gastrointestinal tumors due to its minimally invasive benefits. However, some studies have suggested that, compared to open surgery, laparoscopic surgery may increase the risk of tumor cell dissemination into the peritoneal cavity due to the characteristics of laparoscopic procedures and the effect of pneumoperitoneum (18–20). Moreover, lymphadenectomy may facilitate the spread of tumor cells by opening lymphatic channels (21). These factors could heighten the risk of postoperative PM, particularly after laparoscopic-assisted surgeries. Compared to laparoscopic surgery, robotic surgery improves several critical elements of minimally invasive surgery, including enhanced visualization, a stable view, and superior precision, thanks to articulated instruments and motion scaling (22). These advantages may reduce the risk of tumor cell dissemination by minimizing tissue manipulation and preventing undue pressure on the tumor site. Incorporating robotic surgery could potentially mitigate some of the risks associated with laparoscopic procedures while maintaining the benefits of minimally invasive surgery.

In the search for therapies to prevent postoperative PM in gastric cancer, no satisfactory progress has been made (23). Systemic chemotherapy has demonstrated efficacy in improving prognosis for gastric cancer sufferers (24). However, due to the peritoneal-plasma barrier, intravenously administered drugs struggle to penetrate the peritoneum effectively, resulting in a low efficacy of systemic chemotherapy against PM, with a response rate of only 14% (25, 26). Consequently, increasing attention has shifted toward local treatment strategies, particularly the intraperitoneal infusion of high-concentration chemotherapy agents.

HIPEC enhances local drug concentration by directly infusing high-dose chemotherapy into the peritoneal cavity, effectively targeting and killing microscopic residual tumors or free cancer cells (6). Additionally, hyperthermia can directly cause irreversible damage to cancer cells (27) and effectively enhance the accumulation and toxicity of drugs in cancer cells by increasing drug permeability (28, 29). Furthermore, the mechanical lavage effect of HIPEC can reduce the implantation of free cancer cells on the peritoneal surface (30). These mechanisms synergistically enhance the antitumor effect of HIPEC.

Since Japanese researcher Koga first applied P-HIPEC to prevent postoperative PM in locally advanced gastric cancer in 1988 (31), numerous studies have investigated its role in improving prognosis and reducing PM after radical gastrectomy. Kunte et al. summarized recent studies from international research institutions, the majority of which were from Asia, on the application of P-HIPEC in non-metastatic gastric cancer. They concluded that the combination of P-HIPEC with curative surgery and systemic chemotherapy can lead to significant benefits in DFS (32). In Liu et al.’s study, patients receiving P-HIPEC had a substantially reduced PM rate than those only having surgery (10.5% vs. 27.5%, *P =* 0.015) (15). Similarly, Kang et al. found that the 3-year DFS rate in patients undergoing P-HIPEC treatment was markedly superior to that of the control group (66.03% vs. 28.87%) (33). In our study, the Kaplan-Meier curve before PSM showed higher DFS in the HIPEC group, but the difference was marginally significant (*P =* 0.055), likely due to baseline imbalances between the two groups. After PSM was applied to eliminate confounding factors, DFS in the HIPEC group was noticeably higher than in the control group (*P =* 0.013), and the PM rate was significantly lower (5.3% vs. 17.3%, *P =* 0.039). Our findings are consistent with previous studies, indicating that P-HIPEC can significantly improve DFS in patients. The postoperative PM rate in our study was relatively low, possibly because some patients had concurrent extraperitoneal metastasis at the time of initial PM diagnosis, and these patients were classified into the peritoneal and extraperitoneal recurrence group.

Current research on the impact of P-HIPEC on OS presents mixed findings. Liu et al. reported that the 3-year OS rate in the P-HIPEC group improved (62.7% vs. 48.1%) but the difference did not reach statistical significance (*P =* 0.075). Similarly, the study by Zhong et al. also demonstrated that the 5-year OS rate in the HIPEC group improved but did not reach statistical significance (41.1% vs. 34.5%, *P* = 0.118) (34). In contrast, Kang et al. demonstrated a statistically significant improvement in 5-year OS with P-HIPEC (43.9% vs. 10.7%, *P* = 0.029). In our study, the P-HIPEC group exhibited a higher 5-year OS rate compared to the control group in both the original cohort (55.8% vs. 50.8%) and the PSM cohort (57.6% vs. 50.3%), but these differences did not reach statistical significance (*P* = 0.355 and *P* = 0.215). These findings suggest that while P-HIPEC may confer an OS benefit, the evidence remains inconclusive. Consistent with the analysis of survival rates in the Kaplan-Meier curves, our study’s univariate and multivariate Cox regression analyses demonstrated that P-HIPEC is an independent protective factor affecting patients’ DFS, but not OS. The discrepancy between DFS and OS improvements may be influenced by several factors. To begin with, the relatively small sample size and the limited follow-up period in our study may have insufficient statistical power to detect significant differences in OS. A larger cohort and extended follow-up are essential to observe long-term survival benefits that might emerge over time. In addition, while P-HIPEC is designed to eliminate microscopic residual disease in the peritoneal cavity, it may not exert a significant influence on the incidence of distant metastases in other organs, ultimately resulting in limited overall survival benefits. Finally, as research in gastric cancer molecular biology advances, patients now may benefit from subsequent lines of therapy, including newer chemotherapeutic agents and targeted therapies, which can positively influence OS outcomes despite initial disease recurrence.

Although the efficacy of HIPEC is affirmed in some studies, some researchers have raised concerns about the potential postoperative complications associated with P-HIPEC. Yoshida et al. found that P-HIPEC is linked with risks of myelosuppression, anastomotic leakage, bowel obstruction, or bowel perforation compared to surgery alone (35). However, most recent studies indicate that P-HIPEC does not elevate the risk of postoperative complications (15, 36, 37), aligning with our findings and indicating that P-HIPEC is generally a safe treatment. We believe that advancements in technology and more standardized procedures contribute to the enhanced safety of P-HIPEC. Although no significant differences in common complication rates were observed in our study, the HIPEC group experienced higher rates of bowel obstruction (10.0% vs. 7.1%) and lung infection (8.0% vs. 5.5%) compared to the non-HIPEC group. And the late HIPEC group had a higher incidence of wound infections (6.3% vs. 0%) compared to the early HIPEC group. These trends may be related to increased irritation of the intestines and a higher number of wound exposures, necessitating further clinical observation in the future.

Furthermore, the absence of high-quality, evidence-based guidelines has hindered the establishment of a standardized protocol for P-HIPEC. This has resulted in inconsistencies in clinical practice, particularly concerning HIPEC frequency, initiation timing, and the selection of chemotherapy agents, all of which are also influenced by the patient’s postoperative recovery and tolerance. Current research on determining the optimal number of HIPEC sessions remains quite limited. Zhang et al. found that the choice between single or multiple HIPEC sessions had no significant impact on DFS, OS, or the incidence of postoperative complications (38). Our study reached similar conclusions, showing that the frequency of HIPEC had no significant impact on its efficacy and safety. A plausible explanation is that a single, properly timed and optimally dosed HIPEC session may suffice to eradicate residual peritoneal tumor cells. This suggests that excessive HIPEC may be unnecessary in clinical practice, as it can burden patients without providing additional benefits. Regarding the initiation timing of HIPEC, the consensus is that initiating HIPEC as early as possible is crucial (39). Research indicates that the proliferative kinetics of residual cancer cells change within 24 hours postoperatively, with residual G0-phase cancer cells beginning to enter the proliferative phase. After three days, the proliferation rate slows, and by one week, it returns to preoperative levels (40). All patients in our study began HIPEC within one-week post-surgery, and no significant differences in efficacy and safety were observed between patients who started HIPEC on the day of surgery or within 1-2 days (early HIPEC) and those who initiated it on postoperative day three or later (late HIPEC). The possible reason is that the residual malignant cells had not yet extensively proliferated or established peritoneal colonization during the early postoperative period, ensuring that initiating HIPEC at any point within one week offered a similarly effective therapeutic window. This indicates that it is reasonable to choose different timing for HIPEC based on individual postoperative conditions, rather than adhering to a fixed schedule. It should be noted that our sample size was relatively small, and the stratification of patients by HIPEC frequency and timing further reduced the number of patients in each subgroup, potentially limiting the statistical power to detect subtle differences in outcomes.

Treatment strategies for locally AGC have advanced significantly in recent years. The efficacy of preoperative neoadjuvant chemotherapy has been confirmed by multiple studies and has become an essential component of the standard treatment regimen for locally AGC (41–43). The efficacy and safety of P-HIPEC in patients who have undergone preoperative neoadjuvant chemotherapy remain subjects for further research. At the same time, several prospective randomized controlled trials (NCT02356276, ChiCTR1900024552, NCT01882933) are currently exploring the safety and efficacy of P-HIPEC. The preliminary results from these studies are encouraging and worth anticipating. We look forward to the possible future emergence of a ‘quadruple therapy’—combining neoadjuvant chemotherapy, radical surgery, prophylactic HIPEC, and postoperative adjuvant chemotherapy—that could offer improved prognoses for patients with locally AGC.

There are several limitations to this study. Firstly, it is a single-center retrospective investigation, which inherently carries the risk of selection bias. Factors such as variations in patient demographics, evolving indications for P-HIPEC, and changes in surgeons’ experience could influence the outcomes and introduce biases that PSM cannot fully mitigate. Secondly, we may have overlooked a small number of patients with occult peritoneal metastasis, as not all patients in our study underwent peritoneal cytology. In addition, our relatively small sample size restricts some further analysis within certain groups, such as evaluating the role of P-HIPEC in patients with different levels of adjuvant chemotherapy completion, and prevents the use of PSM to balance baseline characteristics among patients stratified by HIPEC frequency and timing. Ultimately, some patients in our study had limited follow-up duration, which emphasizes the necessity for continued monitoring of these patients. We will also conduct large-scale, prospective, multicenter randomized controlled trials in the future to validate our conclusions.

Overall, our study demonstrates that P-HIPEC can prevent postoperative PM, improve DFS, and not increase the occurrence of postoperative complications in patients with locally AGC, while it does not significantly impact OS. Additionally, the frequency and timing of P-HIPEC initiation appear to have no effect on patient prognosis or the occurrence of postoperative complications.

## Data Availability

The raw data supporting the conclusions of this article will be made available by the authors, without undue reservation.
